# Size-dependent mechanical behavior of nanoscale polymer particles through coarse-grained molecular dynamics simulation

**DOI:** 10.1186/1556-276X-8-541

**Published:** 2013-12-21

**Authors:** Junhua Zhao, Shijo Nagao, Gregory M Odegard, Zhiliang Zhang, Helge Kristiansen, Jianying He

**Affiliations:** 1 NTNU Nanomechancial Lab, Department of Structural Engineering, Norwegian University of Science and Technology (NTNU), 7491 Trondheim, Norway; 2 Department of Mechanical Engineering - Engineering Mechanics, Michigan Technological University, 1400 Townsend Drive, Houghton, MI 49931 USA; 3 Conpart AS, 2013 Kjeller, Norway

**Keywords:** Size effect, Polyethylene particles, Coarse-grained molecular dynamics simulations

## Abstract

Anisotropic conductive adhesives (ACAs) are promising materials used for producing ultra-thin liquid-crystal displays. Because the mechanical response of polymer particles can have a significant impact in the performance of ACAs, understanding of this apparent size effect is of fundamental importance in the electronics industry. The objective of this research is to use a coarse-grained molecular dynamics model to verify and gain physical insight into the observed size dependence effect in polymer particles. In agreement with experimental studies, the results of this study clearly indicate that there is a strong size effect in spherical polymer particles with diameters approaching the nanometer length scale. The results of the simulations also clearly indicate that the source for the increases in modulus is the increase in relative surface energy for decreasing particle sizes. Finally, the actual contact conditions at the surface of the polymer nanoparticles are shown to be similar to those predicted using Hertz and perfectly plastic contact theory. As ACA thicknesses are reduced in response to reductions in polymer particle size, it is expected that the overall compressive stiffness of the ACA will increase, thus influencing the manufacturing process.

## Background

**Figure 1:**
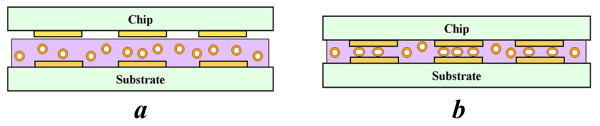
**Compression of polymer particles in anisotropic conductive adhesives. (a)** Before bonding and **(b)** after bonding [2].

For decades, micron-sized spherical polymer particles with well-controlled narrow-size distributions have been used in the pharmaceutical and biotechnology industries. Renewed interest in these particles has been focused on their use in microelectronic devices [1-3]. One of the most promising applications is anisotropic conductive adhesives (ACA) employed for producing ultra-thin liquid-crystal displays, as shown in Figure 1[3-6]. The use of polymer particles in ACAs contributes to reduced package sizes, assembly temperatures, environmental compliance, and manufacture costs. Because the polymer particles used in ACAs can be subjected to large compressive stresses (typically exceeding 30%) during the manufacturing process and in-service operation, it is important to understand the influence of large compressive stresses on their mechanical integrity and performance.

Experimental research has been previously conducted to determine the mechanical response of micron-sized polymer particles by Zhang et al. [5-7]. They used a nanoindentation-based flat punch method to test the compressive response of polymer particles with diameters ranging from 2.6 to 25.1 μm. They observed that decreasing particle diameters resulted in increasing stiffness of the constituent polymer material [6]. Although this type of size effect has been well-documented in crystalline, inorganic materials [8-14], it has not been carefully studied in organic, amorphous materials. The observed behavior of the polymer particles was explained by He et al. [5,6] using a core-shell argument. That is, there exists a layer of polymer at the surface of the particles that has a molecular structure that differs from that found in the bulk polymer (toward the center of the particle). This surface layer has a constant thickness, regardless of the size of the particle. The presence of this surface layer has a diminishing influence on the overall mechanical response of the particle for increasing particle sizes. Although this explanation is plausible, it remains unverified. Because the mechanical response of the polymer particles can have a significant impact on the performance of ACAs, understanding of this apparent size effect is of fundamental importance in the electronics industry.

The objective of this research is to use a coarse-grained molecular dynamics model to verify and gain physical insight into the observed size-dependence effect in polymer particles. Three different types of analyses have been performed to accomplish the objective. First, simulations of the loading sequence of polymer nanoparticles under compression are presented. This is followed by a description of simulations of the unloading process, both of which serve to verify the previous experimental observations. Finally, a surface energy analysis is described where the surface energy is determined for different sizes of nanoparticles to provide physical insight into the size-dependence effect.

## Main text

### Spherical particle molecular models

Although polymer particles can be composed of a wide range of polymer chemistries, linear polyethylene (PE) was chosen as the model material for this study because of its simple conformational structure and the availability of coarse-grained (CG) potentials especially tuned for the surface tension [15]. Zhao et al. [16] previously demonstrated that the CG models are able to effectively capture the thermo-mechanical characteristics of PE in its glassy phase. Well-tuned CG models can be simulated with significantly less time than all-atom models and are especially advantageous for modeled molecular systems with large numbers of atoms. Because of the relatively large size of the simulated systems in this study, a CG modeling technique using LAMMPS molecular dynamic simulation code was adopted based on a semi-crystalline lattice method for generating entangled polymer structures [16-18].

**Figure 2:**
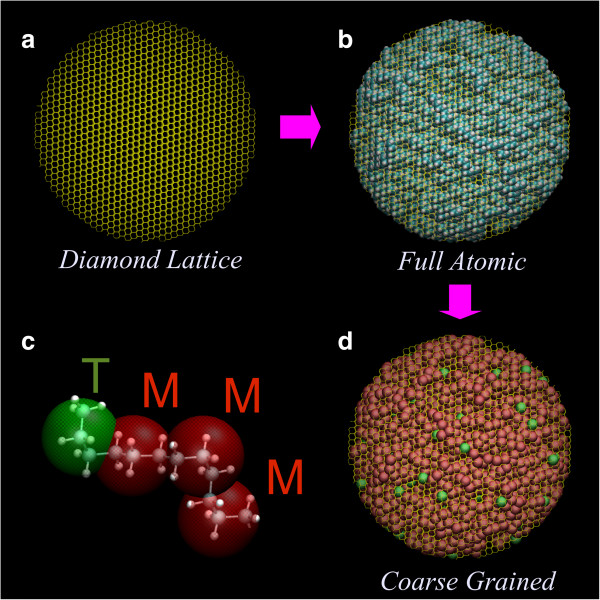
**Coarse-grained (CG) molecular modeling of PE nano-particles using the semi-crystalline lattice method. (a)** The template diamond lattice, **(b)** all-atom model generated by a random walk process on the lattice, **(c)** CG model with terminal (T) and non-terminal (M) beads, and **(d)** final CG model.

The CG modeling process started with the construction of the spherical diamond lattice with a lattice spacing of 0.154 nm (Figure 2(a)). The PE molecules were placed on randomly selected lattice points and then expanded by self-avoiding random walks until the molecules reached a minimum length threshold. A few steps of backtracking were occasionally performed to prevent molecules under this threshold from colliding with neighboring molecules or the surface of the particle. In cases when there was not enough room to achieve the required molecular length after a specified number of trial processes, the molecule was simply discarded. The resulting highly entangled molecular model is shown in Figure 2(b). The model had a relatively uniform density distribution. The molecular model was then converted to a CG bead model where each bead represented three monomer units of PE (Figure 2(c)). As indicated in Figure 2(c), each terminal bead T (marked in green) represented a CH_3_-[CH_2_]_2_ group, while each non-terminal bead M (marked in red) represented a [CH_2_]_3_ group. The resulting CG model of the spherical particle is shown in Figure 2(d).

The CG potential set for PE that was used herein is based on the work of Nielsen et al. [15] for predicting bulk density and surface tension of n-alkanes at room temperature and ambient pressure and is useful for the prediction of thermo-mechanical properties over the glass transition [16]. All the potential parameters used in this study are summarized in Table 1.

**Table 1:** Potential functions and corresponding parameters of coarse-grained method

**Interaction**	**Form**	**Parameters**	**Unit**
Bond	$E=\frac{1}{2}k_{b}{\left(r_{b}-r_{0}\right)}^{2}$	*k*_b_ = 6.96 (TT), *k*_b_ = 6.16 (TM, MM)	kcal/mol Å^2^
		*r*_0_ = 3.65 (TM), *r*_0_ = 3.64 (MM)	Å
Angle	$E=\frac{1}{2}k_{\theta }{\left(\theta -\theta \right)}^{2}$	*k*_θ_ = 1.09 (TMT), *k*_θ_ = 1.19 (TMM, MMM)	kcal/mol
		*θ*_0_ = 175.5 (TMT), *θ*_0_ = 175 (TMM), *θ*_0_ = 173 (TMM)	Degree
Non-bonded	$E=\left(\frac{27\epsilon }{4}\right)\left[{\left(\frac{\sigma }{r}\right)}^{9}-{\left(\frac{\sigma }{r}\right)}^{6}\right]$	*ϵ* = 0.469 (TT), *ϵ* = 0.444 (TM), *ϵ* = 0.42 (MM)	kcal/mol
		*σ* = 4.585 (TT), *σ* = 4.5455 (TM), *σ* = 4.506 (MM)	Å
		*r*_c_ = 15 Å (truncation radius)	
Carbon-CG bead	$E=-\frac{A}{r^{6}}$	*A* = -583.81 (CT, CM)	kcal/mol
		*r*_c_ = 10 Å (truncation radius)	

T is a CH_3_-CH_2_-CH_2_- bead, and M is a -CH_2_-CH_2_-CH_2_- bead. The potentials (CT and CM) between carbon atom and CG bead are for the contact of the polymer particle with the loading plates.

This process was used to construct five different polymer particles with different diameters ranging from 5 to 40 nm, indicated symbolically as *D*_5_ through *D*_40_. The specific details of each of the five particles are listed in Table 2. The largest particle contained over 0.4 million CG beads corresponding to about 3.6 million atoms. Once the initial molecular structure of the CG models was established, each CG model was equilibrated for 200 ps in vacuum at *T* = 500 K using the Nosé-Hoover temperature thermostat and pressure barostat [19]. After the equilibration process, the model particles were cooled down to 250 K, which is slightly lower than the glass transition temperature (280 K) of PE [16]. The resulting average density of the models was 0.836 g/cm^3^, showing a good agreement with the bulk density of linear PE (0.856 g/cm^3^) found in the literature [16,20,21].

**Table 2:** Characteristics of coarse-grained linear polyethylene particles

**Model name**	***D***_**5**_	***D***_**10**_	***D***_**20**_	***D***_**30**_	***D***_**40**_
Number of CG beads	800	6,400	51,200	172,800	409,600
Number of molecules	4	23	256	864	2048
Diameter (nm)	5.00	10.13	20.40	30.09	40.33
Density (g/cm3)	0.854	0.822	0.805	0.846	0.833
Loading step per 20 ps (pm)	3.125	6.250	12.50	18.75	25.00

**Figure 3:**
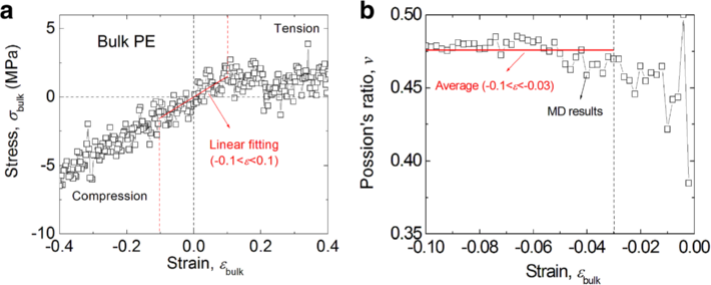
**Mechanical response of bulk PE. (a)** Bulk PE under simulated uniaxial tension and compression; and **(b)** Poisson's ratio of bulk PE under simulated compression.

For comparison purposes, a bulk CG model of linear PE was constructed using the same potential function. The model-building process of this bulk structure was similar to that of the particles, except that the template lattice was shaped in a cubic cell with three-dimensional periodic boundary conditions. After the same annealing process used for the spherical particles, the periodic cluster containing 20,000 CG beads reached the equilibrium simulation box dimensions of 11.8 × 11.8 × 11.8 nm^3^. Simulated uniaxial compression and tension deformations were applied to this model to determine the bulk elastic properties of the PE material. Figure 3 shows the virial stress-strain response from these simulations and the Poisson's ratio for compressive strains. The Young's modulus *E* of the material was calculated to be around 20 MPa for the strain range -0.1 ≤ *ϵ* ≤ 0.1, and the Poisson's ratio *ν* was averaged as 0.476 for the strain range -0.1 ≤ *ϵ* ≤ -0.03.

### Simulated compression loading

**Figure 4:**
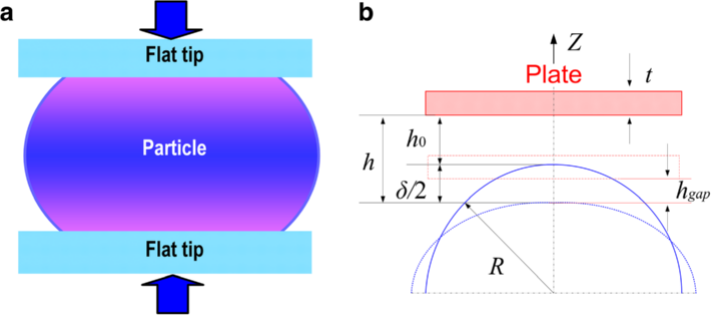
**Applied compression using plate above and below the particles, and dimensions of the compression simulation. (a)** Compression of polymer nanoparticle between two flat, rigid surfaces and **(b)** the dimensions associated with the model.

Simulated compression loadings were performed for each of the particles described in ‘Spherical particle molecular models’ section to determine the influence of particle size on the mechanical response. These simulations are similar to the type of compression loads experienced by polymer particles in ACAs when they are compressed between the flat faces of the contacts between the chip and substrate (Figure 1). The compression was applied to the simulated particles using rigid plates above and below the particles (Figure 4a). Figure 4b shows the dimensions associated with the compression simulations for a spherical particle of radius *R*.

**Figure 5:**
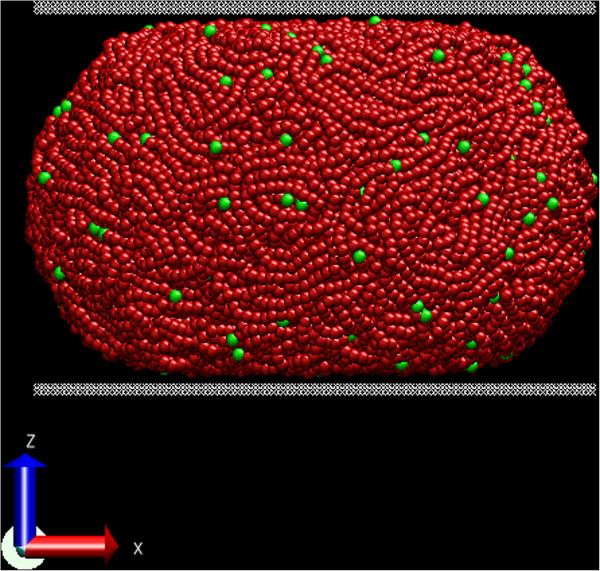
**Compressed configuration of the *D***_**20 **_**spherical particle.**

Computational compression tests of the modeled particles are performed by MD as illustrated in Figure 5. Two diamond plates of thickness *t* = 0.5 nm were placed at both the top and bottom of the particles with a gap of *h*_0_ = 1.0 nm. Constant strain-rate loading was simulated by stepping both the plates towards the particle center, followed by structural relaxation period of 20 ps. Strain rates of 3.125 × 107 s^-1^ were maintained for all particle sizes by adjusting the step distance of the loading plates (see Table 2). The temperature of the particles were kept constant by a Nosé-Hoover thermostat at *T* = 250 K, while the carbon atoms in the loading plates were frozen such that the atoms did not have displacements of any kind except as dictated by the controlled vertical compression. The frozen carbon atoms still maintained the usual non-bonded interactions with the particle molecules (Table 1). This modeling process is similar to that used for silicon nanospheres [22]. Figure 5 shows the compression of the *D*_20_ particle.

To quantify the simulated response of the polymer particles compressed by a load of *P*, the nominal strain and nominal stress were defined as, respectively,

(1) $$\epsilon =\frac{h-h0}{R}$$

(2) $$\sigma =\frac{P}{\pi R^{2}},$$

where *h* is the loading plate displacement from the initial contact position *h*_0_ (Figure 4b). It is important to note that although these parameters are not strains and stresses according to their classic tensoral definitions [23], they are used herein as simple scalar measures in a manner consistent with previous studies [5,6]. Because the initial gap distance *h*_0_ is the same as the non-bonded cutoff radius between the rigid plate atoms and the CG beads (Table 1), any displacement of the plate toward the particle results in an axial loading via the repulsive energy potential; thus, there is no loading slack in the system when *ϵ* = 0. It was assumed that the distance between the particle surface and loading plate during the compression, *h*_gap_, was constant due to the repulsive energy potential [22].

The total load *P* applied onto the sphere was evaluated from the stress response within the plate (because of the load balance between the plate and particle) using

(3) $$P=-\sigma_{Pz}A_{P},$$

where *A*_p_ is the area of the plate normal to the *z*-axis (Figure 4b) and *σ*_Pz_ is the component of the virial stress along the *z*-axis. The usual definition of the virial stress [24] can be simplified for the case of the stress along the *z*-axis in the plate as

(4) $$\sigma_{Pz}=-\frac{1}{V_{P}}\left(\sum_{i}^{N_{\mathrm{carbon}}}mv_{\mathrm{iz}}v_{\mathrm{iz}}+\sum_{i}^{N_{\mathrm{carbon}}}\sum_{j}^{N_{\mathrm{bead}}}r_{\mathrm{ijz}}f_{\mathrm{ijz}}\right)\;,$$

where *V*_P_ denotes the volume of the plate, *m* is mass of carbon atom *i*, *v*_*iz*_ the *z*-component of velocity of atom *i*, *r*_*ijz*_ the *z*-component of the displacement vector between the *i*th carbon and *j*th CG bead, *f*_*ijz*_ is the *z*-component of the force between them, *N*_bead_ is the total number of CG beads, and *N*_carbon_ is the total number of carbon atoms in the plate. Because the carbon atoms in the plate were frozen, the velocity terms in Equation (4) were zero-valued. Substitution of Equation (4) into (3) yields

(5) $$P=\frac{1}{t}\sum_{i}^{N_{\mathrm{carbon}}}\sum_{j}^{N_{\mathrm{bead}}}r_{\mathrm{ijz}}f_{\mathrm{ijz}}.$$

In order to effectively evaluate the size effect in the polymer particles, a continuum model of a particle subjected to compressive loading between two flat plates was evaluated with finite element analysis (FEA). Because the size effect observed in polymer nanoparticles does not exist in the classical continuum modeling of materials, the response of the FEA model is independent of size effects and thus serves as an excellent control reference to compare the molecular modeling results with. Axisymmetric quadrilateral elements were used with the ANSYS finite element software package [25]. Contact elements were placed between the surfaces of the sphere and the rigid plate. The Young's modulus and Poisson's ratio values determined from the bulk MD simulations of PE described in ‘Spherical particle molecular models’ section were used in the FEA model. Displacements were applied to the top surface of the model, and the nominal strains and nominal stresses were measured using Equations (1) and (2), respectively. It is important to note that elastic properties were used to simulate a large deformation of the material. Normally, a hyperelastic analysis would be appropriate for such an analysis; however, the linear approximation is sufficient for the current study as a simple baseline comparison to the MD models.

**Figure 6:**
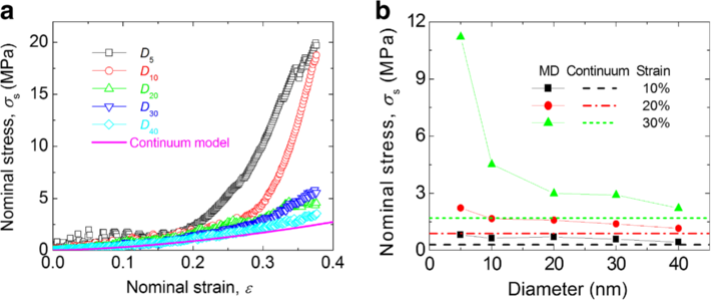
**Particle loading behaviors. (a)** Nominal stress vs nominal strain for five different particle diameters and for the continuum model. **(b)** Nominal stress vs particle diameter for different compressive strain levels.

The nominal stress-strain curves obtained for the MD and FEA simulations are shown in Figure 6a. It is clear that the mechanical responses of the different particles subjected to compressive loading are similar for nominal strains <0.2 and diverge for nominal strains >0.2. Furthermore, it is evident that the smaller the diameter of the nanoparticle, the greater the nominal stress for a given nominal strain >0.2. The lowest stress response belongs to the continuum model, which has no inherent size effect. Therefore, the smaller the particle, the more size effect is exhibited. The larger particles, which have a nominal stress response that approaches that of the continuum model, show decreasing levels of size effect.

Figure 6b displays the particle nominal stresses as a function of particle diameter for different compressive strain levels. For compressive strains of 20%, a mild size effect is observed. At this strain, the nominal stress for the smallest particle is about 1.5 times that of the largest particle. When the compressive strain is increased to 30%, which is common for the micron-sized polymer particles used in ACAs, the nominal stress for the *D*_5_ particle is approximately three times that of *D*_40_ particle. The data in the Figure 6b also indicates that the particle nominal stresses for large particles approach that of the continuum elastic solution.

The size effect data shown in Figures 6 are consistent with the size effect observed experimentally. He et al. [6] carried out experiments on micron-sized polystyrene-co-divinylbenzene (PS-DVB) particles. A nanoindentation-based flat punch method was used to determine the stress-strain behavior of particles in compression. The particle size varied from 2.6 to 25 μm. A strong size effect on the compressive stress strain curve was observed. As the particle size decreases, the mechanical response becomes stiffer.

### Simulated compression unloading

**Figure 7:**
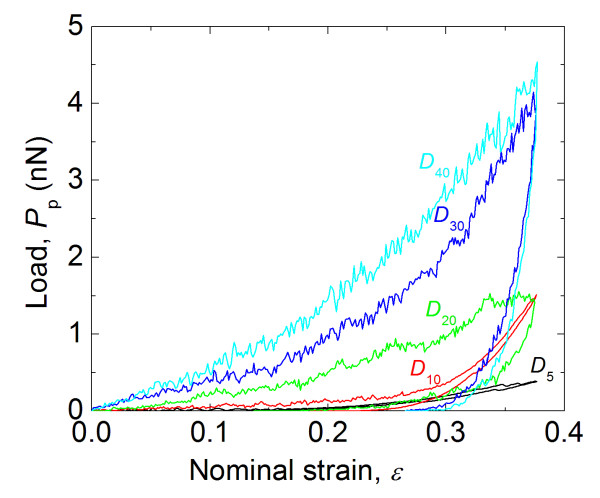
Compressive unloading curves for the five spherical polymer particles.

**Figure 8:**
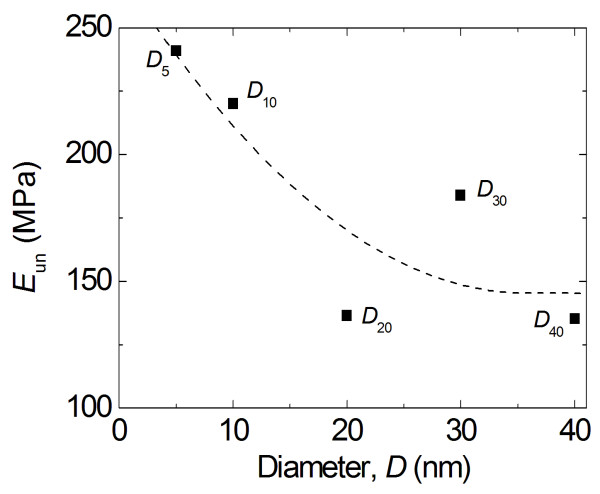
Compressive unloading modulus for each of the five polymer particles.

A series of compression unloading simulations were performed on the same MD models described in ‘Simulated compression loading’ section. The simulated unloadings followed compressive loading strains of 38%. The load-strain diagrams of these simulations are shown in Figure 7. The elastic modulus was determined from the compression unloading curves using [22,26]

(6) $$E_{\mathrm{un}}=\left(\frac{1}{2r_{c}}\right)\frac{dP_{s}}{\mathrm{d\delta}},$$

where *r*_c_ is the contact radius, *P*_s_ is the applied load during unloading, and *δ* is the displacement during unloading. The contact radius was determined from the MD simulations using a method previously developed [26]. The differential term in Equation (6) was determined by fitting the initial unloading *P*_s_-*δ* response with the power function

(7) $$P_{s}=A{\left(\delta -\delta_{f}\right)}^{m},$$

where *A*, *δ*_f_, and *m* are fitting parameters. The calculated elastic moduli are plotted in Figure 8 over the range of diameters of the particles. In general, the data in Figure 8 shows a strong dependence of elastic properties on the particle size, with smaller particles having a stiffer response. This trend is in agreement with the trends observed in Figures 6, which is a supporting evidence for the presence of a significant size effect in polymer particles.

## Discussion

### Surface energy analysis

The results of the compression loading and unloading simulations clearly demonstrate the existence of a size effect in polymer particles. The MD models in this study can also be used to gain physical insight into the origin of the size effect. It is well known that crystalline [27-29] and amorphous materials [30-33] have molecular structures at the surface (or bi-material interface) that differ substantially than in the bulk. In fact, the CG potential used for the research described herein was developed specifically to accurately predict the bulk and surface structure of PE [15]. For amorphous polymers, the above-cited references show that the mass density of the polymer is higher on the surface than in the bulk. This high-density layer has a thickness on the order of 1 nm.

The cause of the densification of polymer molecules on a surface is classically explained by the concept of surface tension. Segments of polymer molecules in the bulk have a relatively low energy state because of the balance of attractive short-range (e.g., covalent bonds) and long-range (e.g., van der Waals bonds) interactions in every direction. Segments of polymer molecules on a free surface (or a non-bonded bi-material interface of two dissimilar materials) do not have these strong attractive interactions in the direction normal to the surface and are thus pulled by the attractive forces in the opposite direction towards the bulk. As a result, there is a densification of the top layer of polymer molecules on a surface.

This densified surface layer of material has a constant thickness regardless of the size and geometry of the overall material structure. For polymer particles, this means that the surface layer will have the same finite thickness for any particle size. For decreasing particle sizes, the relative volume fraction of the densified material increases. Therefore, it follows that the smaller polymer particles studied herein are expected to have stiffer mechanical responses than the larger particles, as observed experimentally [5-7] and discussed in ‘Simulated compression loading’ and ‘Simulated compression unloading’ sections.

**Figure 9:**
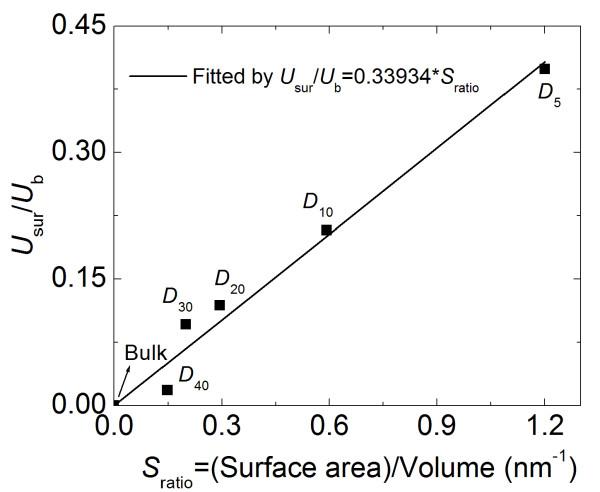
**Normalized surface energy vs ratio of surface area to volume (*S***_**ratio**_ **= 6/*D*).**

In order to quantify the influence of the surface layer on the mechanical response of the polymer particles, the surface energy has been determined for each diameter. The total internal energy associated with the presence of the surface (i.e., surface energy) in a molecular system can be determined by

(8) $$U_{\mathrm{sur}}=U_{b}-U_{\mathrm{particle}},$$

where *U*_particle_ is the total energy (kinetic plus potential) of a polymer particle, and *U*_b_ is the total energy in a bulk sample of material with the same number of CG beads. These potential energies were calculated using the potential shown in Table 1 using the procedures outlined in ‘Spherical particle molecular models’ section. Figure 9 shows a plot of the ratio *U*_sur_/*U*_b_ over the ratio of the surface area to volume for each of the five particles. Therefore, this plot shows the normalized relationship between the relative particle surface area and the observed surface energy. Clearly, there is a linear relationship (curve fit shown) between the surface energy and the relative surface area, reaffirming that the observed surface energy is physically confined to the surface of the particles and that the relative amounts of surface energy increase for decreasing particle sizes.

**Figure 10:**
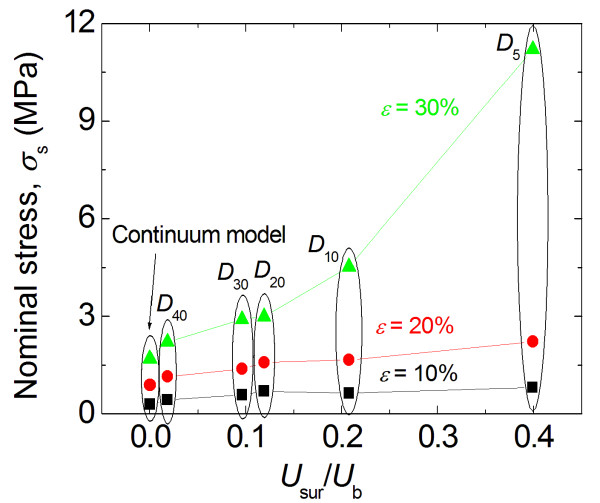
Compressive nominal stress vs normalized surface energy for three compressive strain levels.

**Figure 11:**
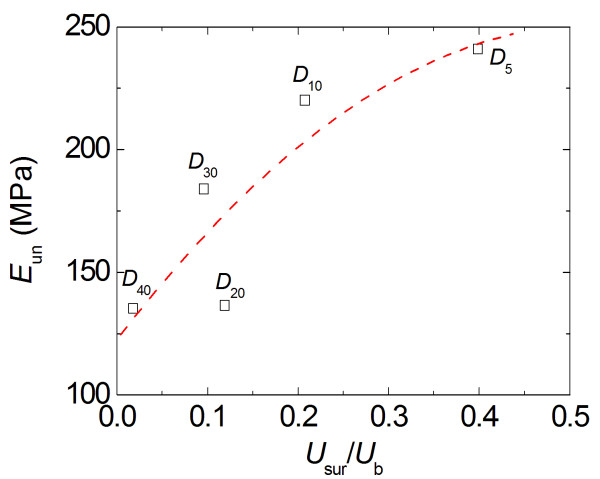
Unloading modulus vs normalized surface energy.

The data plotted in Figures 6b and 8 are replotted with respect to the relative surface energy in Figures 10 and 11, respectively. From Figure 10, it is clear that the nominal compressive stress increases as the surface energy increases (and as the particle size decreases), particularly at higher compressive strains. Figure 11 suggests that the apparent modulus measured from compressive unloading increases with increasing surface energies and decreasing particle sizes. Both Figures 10 and 11 emphasize that decreasing particle sizes result in increases in relative surface energy, which result in increases in particle stiffness. Furthermore, because of the linear relationship between relative surface energy and surface areas shown in Figure 9, it also implies that the compressive nominal stress and unloading modulus will show a similar dependence as a function of surface area.

### Contact radius during compressive loading

The simplest theory for estimating the contact radius during compressive loading is through the Hertz contact theory, which is most suited for linear-elastic materials under compressive strains under 1% [7]. This theory stipulates that the contact radius is calculated by [24]

(9) $$r_{\mathrm{Hertz}}={\left(\frac{1}{2}\mathrm{\delta R}\right)}^{1/2}.$$

For perfectly plastic materials, an alternative approach to determine the contact radius is [24]

(10) $$r_{\mathrm{Geom}}={\left(\mathrm{\delta R}-\frac{\delta^{2}}{4}\right)}^{1/2}.$$

These two approaches are most valid for two extremes in material behavior: linear elasticity and perfect plasticity. However, polymer materials typically exhibit non-linear behavior that is between these two extremes, particularly the PE material considered herein [6]. Therefore, it is important to determine the accuracy of these two simple approaches when applied to polymeric materials.

**Figure 12:**
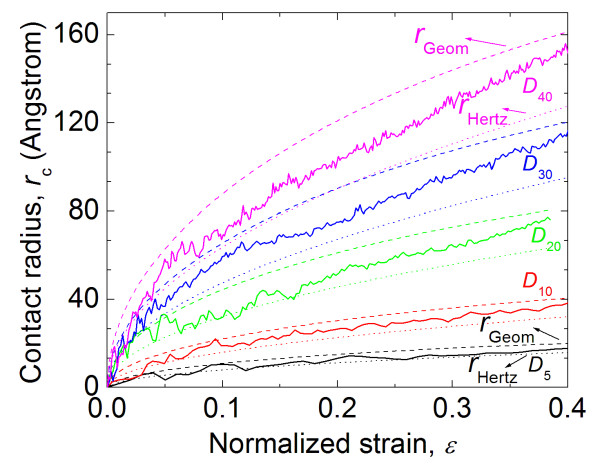
**Contact radius for different particle sizes.** These are from MD simulations (solid lines), Hertz contact theory (dotted lines), and elastic-plastic theory (dashed lines).

In Equation (6), the contact radius was determined directly from inspection of the molecular models as a function of applied compressive strain, similar to an approach used previously [26]. Figure 12 shows this calculated contact radius as a function of nominal strain, and particle size. As expected, the contact radius increases for increasing compressive loads and particle sizes. Also shown in Figure 12 is the contact radii calculated using Equations (9) and (10). These contact radii show the same general trends as the contact radii calculated from MD as a function of nominal strain and particle size. Also, the contact radii calculated from MD are bounded by those calculated from Hertz and perfect plasticity theory. This result is not surprising considering that the elastic-plastic behavior of PE lies between the extremes of linear elasticity and perfect plasticity. It is also evident in the figure that as the compressive nominal strain increases, the material behavior tends to approach that of Hertz contact theory and the perfect plasticity theory. This observation is in good agreement with elastic-plastic FEA simulations [34].

## Conclusion

In agreement with experimental studies [5-7], the results of this study clearly indicate that there is a strong size effect in spherical polymer particles with diameters approaching the nanometer-length scale. As the particle diameter decreases from 40 to 5 nm, increases in elastic modulus are predicted from the molecular simulations. These increases in modulus are significant for compressive nominal strains below 30% and substantially large for strains greater than or equal to 30%. The results of the simulations also clearly indicate that the source of the increases in modulus is the increase in total energy at the surface of the particles, that is, the surface energy. As the particle diameter decreases, the relative surface energy (ratio of surface energy to equivalent bulk energy for the particle volume) increases. The increases in surface energy result from the increases in the mass density of the material at the surface. This local increase in mass density results in an overall increase in particle stiffness properties.

These results are of significant importance for two reasons. First, coated polymer particles used for electrical conduction in ACAs have a very strong size-dependent behavior. As particle sizes are reduced, they will have a stiffer response to the compressive forces, particularly for nominal compressive strains of at least 30%. Therefore, as ACA thicknesses are reduced in response to reductions in liquid-crystal display thicknesses, it is expected that the overall compressive stiffness of the ACA will increase, thus influencing the manufacturing process. Second, these results indicate the presence of very strong size-dependent effects in organic, amorphous nanostructures that have been well-documented for inorganic, crystalline nanostructures, such as nanowires and nanobelts. The size dependence is a direct result of the changes that occur in the structure of the polymer molecules on the particle surface.

## Competing interests

The authors declare that they have no competing interests.

## Authors’ contributions

JZ and SN constructed the coarse-grained polymer model and carried out the simulation. JZ, GO, and JH drafted the manuscript. ZZ and HK initiated and supervised the research work. All authors participated in the analysis of the data, contributed to the discussions, and proofread the manuscript. All authors read and approved the final manuscript.
